# The British Journals: Physiology

**Published:** 1838-10

**Authors:** 


### III. THE BRITISH JOURNALS.

(FOR THE QUARTER ENDING AUGUST 31, 1838.)

### PHYSIOLOGY.

*Observations on the Fluid in the Vesiculæ Seminales of Man.* By JOHN DAVY, M.D. F.R.S., Assistant Inspector of Army Hospitals.

[THIS is an interesting communication, and adds considerably to our knowledge of an obscure subject. Like all the writings of Dr. Davy, it bears the stamp of accurate observation and sober deduction. Its object is to endeavour to settle the difference of opinion existing among physiologists respecting the nature of the fluid of the vesiculæ seminales; viz. "whether it is secreted by the testes or by the vesiculæ; and whether, in consequence, the vesiculæ are to be viewed chiefly as reservoirs or merely as glands?" With the view of throwing light on this subject, Dr. Davy examined the fluid in the vesiculæ and in the vasa deferentia after death, in a variety of cases at the Military Hospital at Chatham, of which he is superintendent. Twenty of these examinations are here detailed. The following extracts exhibit, 1, the inferences respecting the questions in dispute, deducible from the observations; 2, the effects of disease on the spermatic fluid; and, 3, the important bearing of such enquiries on certain questions in medical jurisprudence.]

1. The first inference that appears to me unavoidable is, that the vesiculæ are seminal reservoirs, according to the old opinion on the subject, and that which is still most commonly entertained by the continental physiologists: and next, that they are not merely reservoirs, but are also secreting organs, furnishing mucus, and perhaps some other fluid, for admixture with the semen.

The first inference is supported by the general resemblance, in several cases, of the fluid of the vasa deferentia and of the vesiculæ, and of the existence of the characteristic spermatic animalcules in the fluid of the vesiculæ, in every instance in which they were detected in the fluid of the vasa deferentia. Hunter does not mention having used the microscope in his enquiry. If he had, he could hardly have failed to have arrived at a different conclusion.

The second inference is supported by there being a certain difference in almost every case between the fluid of the vesiculæ and that of the vasa deferentia, and especially by the circumstance that the difference of quality is most perceptible in the fluid of the fundus; where most out of the way of being readily mixed with the fluid of the testes. What the exact difference of qualities is between the fluid of the vesiculæ and of the vasa deferentia, and, it may be added, of the vasa deferentia and of the testes, in perfect health, remains to be ascertained. It can be determined only by careful examination and comparison in the instances of criminals who have been executed, or of persons who have been killed by accident, not labouring under chronic disease, and in the vigour of life. I am disposed to think that the difference will not be found very considerable; and that between the fluid of the vesiculæ and of the vasa deferentia it will consist chiefly in the former being more dilute, and perhaps more bland and mucous. . . .

2. Relative to the effects of disease on the fluid of the vesiculæ seminales, and on the spermatic fluid generally, the instances brought forward are too few to admit of extensive induction. They seem to show, first, that chronic wasting diseases, terminating in death, arrest the secretion of the testes, or the production of those animalcules, on which there is much reason to infer the active power of the semen depends; 2dly, that the contents of the vesiculæ and vasa deferentia, under the influence of disease, retain longer their characteristic qualities than the contents of the tubuli; and, 3dly, that there is least fluid in the vesiculæ and in the vasa deferentia, and that it is most altered in instances of chronic diseases of the abdominal viscera, and especially of the intestines. . . .

3. Admitting that spermatic animalcules are characteristic of and essential to healthy spermatic fluid, in certain doubtful criminal cases, probably decisive evidence may be obtained by means of microscopical examination. The spermatic fluid undergoes change rapidly when exposed to the air, and even soon becomes putrid; but the spermatic animalcules, I find, resist change in a remarkable manner. In one instance distinct remains of these animalcules were observed in putrid fluid, which had been kept ten weeks, at a temperature varying between fifty and sixty degrees of Fahrenheit. In another instance some fluid of the vesiculæ was applied to linen, and wrapped in paper, and put by in a close drawer. It was examined the following day, at the end of a week, and after eighteen days; and each time animalcules were discovered under the microscope. The mode of making the trial was by saturating a small portion of the smeared linen with a few drops of water, and gently pressing out a drop for the experiment. Fragments of the animalcules were very distinct, and sufficiently characteristic; and, on careful inspection, an entire animalcule, here and there, was observed. The application of these facts to the purposes of evidence does not require any comment.

*Edinburgh Med. and Surg. Journal. July* 1, 1828.

---

*An Experimental Enquiry into the Influence of Nitrogen on the Growth of Plants.* By ROBERT RIGG, Esq.

THE author, after briefly alluding to a former paper laid before the Royal Society, describing the chemical changes which occur during the germination of seeds, and some of the decompositions of vegetable matter, proceeds, in the present paper, to trace a connexion between the phenomena exhibited during the growth of plants, and the direct agency of nitrogen. The experiments by which the author supports his views are arranged in separate tables, so drawn out as to indicate not only the quantities of carbon, oxygen, hydrogen, nitrogen, and residual matter, in about 120 different vegetable substances, but also the quantity of nitrogen in each compound, when compared with 1000 parts by weight of carbon in the same substance. The most important of these tables are those which exhibit the chemical constitution of the germs, cotyledons, and rootlets of seeds; the elements of the roots and trunks of trees; and the characters of the various parts of plants, especially of the leaves, at different periods of their growth. From this extensive series, which is stated to form but a small portion of the experiments made by the author in this department of chemical research, it appears that nitrogen and residual matter are invariably the most abundant in those parts of plants which perform the most important offices in vegetable physiology: and hence the author is disposed to infer that nitrogen, (being the element which, more than any other, is permanent in its character,) when coupled with residual matter, is the moving agent, acting under the living principle of the plant, and moulding into shape the other elements. The method of ultimate analysis adopted by the author enables him, as he conceives, to detect very minute errors, and therefore to speak with certainty as to the accuracy and value of every experiment.

*Proceedings of the Royal Society. May* 31, 1838.

---

*Researches on Suppuration.* By GEORGE GULLIVER, Esq., Assistant Surgeon to the Royal Regiment of Horse Guards.

THE author, in consequence of some theoretical views of the suppurative process, was led to undertake an examination of the blood in the different forms of fever accompanying inflammation and suppuration; and the result has been the detection of globules of pus in that fluid in almost every instance where there had existed, during life, either suppuration or great tumefaction of the external parts without the presence of pus. The means by which he detected pus in the blood were partly chemical, and partly by the aid of the microscope. Availing himself of the solvent power which water exerts on the globules of the blood, while it has no action on those of pus, he had merely to dilute the suspected blood sufficiently with water, by which means the red globules were made to disappear, while those of pus remained at the bottom of the fluid, and were easily recognized by a good microscope. A number of cases are detailed, from which the general result above stated was deduced. He considers that his experiments tend to establish the conclusion that suppuration is a kind of proximate analysis of the blood. As the fibrin separated from this fluid produces swelling of the part affected, or is attracted to the contiguous tissue for the reparation of the injury, the globules of the blood, altered by stagnation, become useless, and are discharged as excrementitious matter from the system. Such is the constitution of healthy pus; but, when mixed with broken-down fibrin, it assumes the flaky and curdled appearance, with proneness to putrefaction, characterizing unhealthy pus, and the presence of which in the blood is connected with fevers of the inflammatory or typhoid form.

*Proceedings of the Royal Society. June* 14, 1838.

---

*On the Differences of the Laws regulating Vital and Physical Phenomena.* By WM. B. CARPENTER, M.R.C.S., late President of the Royal Medical and Royal Physical Societies, Edinburgh.

[THIS paper constitutes a portion of the essay to which was recently adjudged the prize annually raised by the contributions of the students, and awarded by the professors of the university of Edinburgh. It is, like all the writings of Mr. Carpenter, marked by views at once clear and comprehensive; and gives still further evidence of the author's remarkable power of handling abstruse physiological subjects at once logically and agreeably. We are happy to learn that Mr. Carpenter has in the press an original work on "General Physiology;" a subject hitherto most imperfectly treated, and one which, we feel assured, will assume a striking degree of interest in his hands, and fill up a blank, not merely in the course of study of the medical man, but in that of men of science generally. We regret extremely that our limits will permit us to lay before the reader no part of the present valuable contribution, except the *general conclusions* deduced by the author from his enquiries: these are as follows:

1. That the *properties* of any aggregation of matter depend upon the method in which its ultimate molecules are combined and arranged.

2. That the simplicity of our notion of the properties of inorganic matter depends upon the facility of our becoming acquainted with them through the command which we possess over the agencies by whose operation they are manifested.

3. That the vital properties of organized tissues are not less the result of their material constitution; but that, whilst the *materials* of an organized tissue may be prepared by the operation of the ordinary laws of affinity acting under peculiar conditions, the tissue cannot be constructed without the agency of a previously existing vitality; and that hence man is debarred from the most advantageous means of becoming acquainted with the laws of physiology.

4. That *vital properties* are not *added* to matter in the process of organization; but those previously existing, and hitherto inactive, are called out or developed.

*Edinburgh New Philosophical Journal. April,* 1838.

